# Anisocoria in a 10-month old girl in the immediate preoperative setting: can you proceed with surgery?

**DOI:** 10.1016/S1674-8301(11)60030-4

**Published:** 2011-05

**Authors:** Zoel A Quinonez, Niroop R Ravula

**Affiliations:** Department of Anesthesiology and Pain Medicine, University of California Davis Health System, CA 95817, USA

**Keywords:** anisocoria, anticholinergics, ipratropium

## Abstract

We report the case of a 10-month old girl with a significant past medical history who presented for elective surgery with a new-onset fixed, dilated pupil. We briefly review the diagnostic approach to such patients and provide guidelines for managing these patients in the immediate preoperative setting.

## CASE REPORT

A 10-month old girl ventilated *via* tracheostomy was transferred from the pediatric intensive care unit (PICU) to the preoperative area for her elective esophagogastroduodenoscopy (EGD) and dilation for esophageal stenosis. Her past medical history included several repaired congenital cardiac and midline defects (truncus arteriosus, right pulmonary artery stenosis, tracheoesophageal fistula, and a cleft lip) and she received nutrition through a gastrostomy tube due to esophageal stenosis, for which she received frequent dilations. She presented with a new-onset fixed and dilated left pupil but had no other focal neurologic deficits or change from her baseline condition. She had a briskly reactive right pupil. Given her medical history and congenital cardiac disease, the anesthesia, surgery and PICU team decided to delay the case to obtain a head computerized tomography (CT) scan before proceeding with anesthesia. The CT scan showed no findings indicative of lesion, thrombus or inflammation. The patient remained stable, returned to the operating room later in the day and underwent her EGD and dilation under general anesthesia without complications. After reviewing the patient's record and deliberating with all members of the PICU team, we found that ipratropium bromide metered dose inhaler (MDI) administered by the patient's nurse before bringing her to the operating room was the only possible causative agent for her anisocoria. The mydriasis resolved by the next morning and no further episodes were noted.

## DISCUSSION

Inhaled anticholinergics are bronchodilators widely used in the inpatient setting, in particular, the intensive care unit. While relatively safe, side effects such as dry mouth, bitter taste and coughing, are well known[1]. Mydriasis from accidental ophthalmic administration of nebulized ipratropium[2]–[5], and rarely from a MDI[6],[7], has also been described.

There been also have reports of acute angle-closure glaucoma (AACG) from accidental ophthalmic administration of ipratropium[8], generally in combination with a β-2 receptor agonist[9]–[14]. The mechanism for AACG includes mydriasis with decreased aqueous humor outflow from the anticholinergic properties of ipratropium, combined with increased aqueous humor production caused by β-2 receptor agonists[9]–[11]. Patients with narrow-angle glaucoma or an undiagnosed narrow angle are at greatest risk of developing AACG[10],[11].

Here, we address for the first time anisocoria presenting in the immediate preoperative period. The potential cost to the patient and institution of unnecessarily cancelling or delaying surgery, and that of a potentially needless workup, underscores the importance of approaching the unilaterally fixed and dilated pupil in a systematic way. In this case, we delayed surgery given this patient's significant past history, but in retrospect realized that some simple steps may have prevented the cost of lost OR time and the additional imaging.

Thus, we suggest some guidelines for deciding whether or not to proceed with elective surgery when confronted with new-onset unilateral mydriasis. These guidelines focus on stratification of patients into those requiring urgent versus non-urgent workup. The first step is to look for associated symptoms and to take a detailed history[15], with the understanding that this poses a particular challenge in small children. If the patient has an altered level of consciousness, an altered mental status, or evidence of direct damage to the eye, an urgent workup should be pursued. Additionally, if there is a history of glaucoma or a congenital narrow angle, the case should be cancelled to seek further workup. Lastly, if there are concerning symptoms, such as eye pain, ophthamloplegia, a frontal headache, or a change in visual acuity, while possibly benign[16]–[18], concern for AACG warrants further investigation in lieu of a non-emergent surgery.

In the setting of no associated symptoms, a detailed history will separate a possible pharmacologic cause, such as ipratropium or accidental ophthalmic scopolamine administration[19],[20], from other causes like a third cranial nerve palsy or damage to the third cranial nerve[15]. If drug exposure is unclear from the history, 1% ophthalmic pilocarpine or convergence of the eye can be used to distinguish between a pharmacologic etiology and a third nerve palsy[15],[19]. Ophthalmic administration of pilocarpine, a muscarinic agonist, will constrict the circular muscles of the iris and cause miosis in the setting of available receptors. If ipratropium, or scopolamine (an antimuscarinic), has been administered to the eye, receptor blockade will prevent constriction of these circular muscles[15],[19]. Of note, low dose pilocarpine, such as the 0.1% formulation, will not cause constriction in the setting of mydriasis from a third nerve compression, and can lead to incorrect, and potentially dangerous, classification of a third nerve compression injury as mydriasis resulting from local application of an anticholinergic or antimuscarinic[19].

In an adult or a child who is able to follow commands, convergence of the eyes will yield the same result, convergence leading to circular muscle constriction and subsequent miosis in the absence of pharmacologic blockade[15],[19]. If a pharmacologic etiology is confirmed, and there exists little concern for AACG, that is, the patient does not have a history of glaucoma or a known shallow anterior chamber and narrow angle, the team can proceed with surgery. A third nerve palsy, on the other hand, warrants case cancellation, an ophthalmology consultation and further workup.

Inpatients with suspected pharmacologic anisocoria who undergo surgery should be monitored for its resolution. If the mydriasis fails to be resolved within a day, or if the patient develops concerning symptoms at any time in the post-operative period, consultation with an ophthalmologist should be immediately obtained. In the case of an ambulatory procedure, the patient and the patient's family should be instructed on where to follow up if the patient develops concerning symptoms or if the anisocoria fails to be resolved within a day.

In summary, anesthesiologists in the preoperative area decide whether a patient can safely proceed to surgery. Failing to take a systematic approach to the preoperative assessment can lead to either inappropriately proceeding with surgery or delaying a crucial intervention, both of which have cost implications for patients and hospitals. In the setting of a new-onset unilateral mydriasis, steps can be taken to determine whether or not to proceed. In this instance, cancellation was not warranted and following these outlined steps would have prevented unnecessarily delaying the case.

## References

[1] Gross NJ (2006). Anticholinergic agents in asthma and COPD. Eur J Pharmacol.

[2] Cabana MD, Johnson H, Lee CK, Helfaer M (1998). Transient Anisocoria secondary to nebulized ipratropium bromide. Clin Pediatr.

[3] Geraerts SD, Plötz FB, van Goor C, Duval EL, van Vught H (2000). Unilateral fixed dilated pupil in a ventilated child with asthma. Eur J Emerg Med.

[4] Udy A (2005). A 10-year-old child with status asthmaticus, hypercapnia and a unilateral dilated pupil. Pediatr Anesth.

[5] Wehbe E, Antoun SA, Moussa JK, Nassif II (2008). Transient Anisocoria caused by aerosolized ipratropium bromide exposure from an Ill-Fitting face mask. J Neuroophthalmol.

[6] Bond DW, Vyas H, Venning H (2002). Mydriasis due to self-administered inhaled ipratropium bromide. Eur J Pediatr.

[7] Weir RE, Whitehead DE, Zaidi FH, Greaves BB (2004). Pupil blown by a puffer. Lancet.

[8] Malawi JT, Robinson GM, Seneviratne H (1982). Ipratropium bromide induced angle closure glaucoma. N Z Med J.

[9] Fernandez-Barrientos Y, Jimenez-Santos M, Martinez De-La-Casa JM, Mendez-Hernandez C, Garcia-Feijoo J (2006). Acute angle-closure galucoma resulting from treatment with nebulised bronchodilators. Arch Soc Esp Oftalmol.

[10] Kalra L, Bone MF (1988). The Effect of Nebulized Bronchodilator therapy on intraocular pressures in patients with glaucoma. Chest.

[11] Lachkar Y, Bouassida W (2007). Drug-induced acute angle closure glaucoma. Curr Opin Ophthalmol.

[12] Mulpeter KM, Walsh JB, O'Connor M, O'Connell F, Burke C (1992). Ocular hazards of nebulized bronchodilators. Postgrad Med J.

[13] Packe GE, Cayton RM, Mashhoudi N (1984). Nebulised Ipratropium bromide and salbutamol causing closed-angle glaucoma. Lancet.

[14] Singh J, O'Brien C, Wright M (1993). Nebulised bronchodilator therapy causes acute angle-closure glaucoma in predisposed individuals. Respir Med.

[15] Lee AG, Taber KH, Hayman LA, Tang RA (1997). A Guide to the isolated dilated pupil. Arch Fam Med.

[16] Pokhrel PK, Loftus SA (2007). Ocular Emergencies. Am Fam Physician.

[17] Jacobson DM (1995). Benign episodic unilateral mydriasis. Clinical characteristics. Ophthalmology.

[18] Manai R, Timsit S, Rancurel G (1995). Unilateral benign episodic mydriasis. Rev Neurol.

[19] Moeller JJ, Maxner CE (2007). The Dilated Pupil: An Update. Curr Neurol Neurosci Rep.

[20] Rubin MM, Sadoff RS, Cozzi GM (1990). Unilateral mydriasis caused by transdermal scopolamine. Oral Surg Oral Med Oral Pathol.

